# Large eddy simulation of dispersion of hazardous materials released from a fire accident around a cubical building

**DOI:** 10.1007/s11356-021-13604-3

**Published:** 2021-05-06

**Authors:** Konstantinos Vasilopoulos, Ioannis Lekakis, Ioannis E. Sarris, Panagiotis Tsoutsanis

**Affiliations:** 1 Centre for Computational Engineering Sciences, Cranfield University, College Road, Cranfield, MK43 0AL UK; 2 Department of Mechanical Engineering, University of West Attica, Athens, Greece

**Keywords:** LES, Smoke dispersion, Fire accident, Toxic zones

## Abstract

The turbulent smoke dispersion from a pool fire around a cubical building is studied using large eddy simulation at a high Reynolds number, corresponding to existing experimental measurements both in laboratory and field test scales. Emphasis of this work is on the smoke dispersion due to two different fuel pool fire accident scenarios, initiated behind the building. For the setup of fire in the first case, crude oil was used with a heat release rate of 7.8 MW, and in the second, diesel oil with a heat release rate of 13.5 MW. It is found that in both fire scenarios, the downstream extent of the toxic zone is approximately the same. This is explained in terms of the fact that the smoke concentration and dispersion are influenced mainly by the convective buoyant forces and the strong turbulence mixing processes within the wake zone of the building. It is suggested that wind is the dominating factor in these accident scenarios, which represent the conditions resulting in the highest toxicity levels.

## Introduction

Certain human activities could in some cases influence significantly the natural atmospheric environment system with disastrous consequences. In such activities, accidental release of hazardous particles and air pollutants are included causing damages to the ecosystem and, also, short- or long-term adverse effects on human health (i.e., cancer, poisonous gas effects on blood, onset of lung’s cancer, acute pneumopathies, and even death). These releases could come from various sources such as the transportation sector (i.e., cars, planes, trains), the industrial sector (i.e., electrical energy production based on the use of fossil fuels), and human actions. A substance is characterized as hazardous if it could put at risk human life. Scientists have shown that it is rather difficult to identify and prevent a hazardous incident by acting effectively in order to minimize its risks (Schnepp et al. 2009). The hazard assessment methodology identifies systematic hazards, records their causes, and suggests protection measures (Argyropoulos et al. 2012). This methodology is based on a risk analysis process that determines the concentration levels of hazardous materials and the corresponding safety limits (Argyropoulos et al. 2010) which, in turn, define danger zones ending up with a risk map.

Accidents of hazardous substances released could be studied at different urban scales in order to decrease possible danger on humans and property. Although hazardous releases may cover a wide urban area, their most devastating effects will be very close to their source of release (Vasilopoulos et al. 2018). In the scenarios of fire, the flow is driven by buoyant forces that increase the turbulent mixing in the rising plume (Hoffmann and Markatos 1988). For this purpose, we believe that it is essential to understand the mechanisms of hazard dispersion (mainly concerning the poisonous gases and ultrafine smog particles) due to buoyancy, turbulent transport, and wind flow effects close to the released material sources and around a simplified structural geometry. Laboratory and field experiments as well as numerical simulations with the use of computational fluid dynamics (CFD) tools are the most suitable methods to deal with the analysis of such hazardous released substances. Considerable work is devoted to experimental studies related to the pollutant dispersion around cubical geometries in wind tunnels (Li and Meroney 1983; Robins and Castro 1977; Thompson 1993; Thompson and Lombardi 1977; Zhang et al. 1996), and on real field experiments studying the effects of wind flow (Richards and Hoxey 2006; Richards and Hoxey 2008) and pollutant dispersion around cubical geometries (Mavroidis et al. 1999; Ogawa and Oikawa 1982). Wind tunnel experiments result in larger turbulent fluctuations and buoyant plumes in comparison to corresponding field experiments (Higson et al. 1996).

Due to the difficulty involved in performing laboratory and field experiments, the pollutant dispersion around cubical geometries is investigated mostly using numerical simulations (Delaunay et al. 1997; Meroney et al. 1999; Rossi et al. 2010; Tominaga and Stathopoulos 2009; Tominaga and Stathopoulos 2010; Vasilopoulos et al. 2019). Even though CFD methods are computationally demanding, they can model the equations of fluid motion and heat/mass transfer in order to provide accurate predictions of accidental release of hazardous substances (Argyropoulos et al. 2010). The flow around a cube is more accurately calculated using the large eddy simulation method (LES) which solves explicitly the momentum transport equation (Breuer et al. 1996; Lim et al. 2009; Richards and Norris 2015; Rodi 1997) predicting at a more efficient level the turbulent flow characteristics.

Several studies exist for the fire and pollutant dispersion/mechanism in an open-air atmospheric environment (Ahn et al. 2019; Zhang et al. 2014). A fire accident around a building creates buoyant forces due to extremely high heat loads that are released instantly (Hu et al. 2009; Pesic et al. 2014; Zhang et al. 2019). Just a few papers focus on the study of the pollutant dispersion behind a cube at different Froude numbers (Zhang et al. 1996) and fluids subjected to different buoyant forces (Tominaga and Stathopoulos 2018). Olvera et al. (2008) studied numerically the buoyant and the neutral plume dispersion within the recirculation cavity of a cube. Olvera and Choudhuri (2006) carried out comparative studies of the emission source position on the surroundings by placing it upstream and downstream of the cube geometry. They concluded that the greatest effect is produced when the source emission is located in the wake area of the cube.

Up today, none of the above studies have been referred to or described the danger area, which is created around a building at the instance of a fire accident. In this study, the smoke’s toxic zone in the wake area of a cubical building is carefully examined in order to mark the toxic limits that prevent harmful outcomes (safe zone). Therefore, for this work, an accurate LES model is used along with an unstructured mesh, suitable for the complex urban terrain, in order to predict the flow pattern and the hazardous dispersion characteristics around a cubical building taking into account that the fire accident is occurring at the wake zone of the reference building.

In order to achieve the aforementioned objectives, (a) the flow numerical results around a cube without hazardous materials are compared with the experimental data of the SILSOE cube (Richards and Hoxey 2012), (b) two fuel pool fire accident scenarios are studied, a crude and a diesel oil one, and (c) the smoke distribution is computed and the toxic zones around the cube are defined. The smoke dispersion from the buoyant forces is compared with the measurements of Tominaga and Stathopoulos (2018).

The novelty of this work is that for the first time, accurate prediction and description of the risk zones created by different pool fire accidents in the wake zone of an isolated building are both made using the LES method. Furthermore, the mechanisms responsible for the smoke dispersion inside the wake zone due to the convective and turbulent concentration fluxes are analyzed.

## Configuration and smoke dispersion modelling

### Problem configuration

**Fig. 1:**
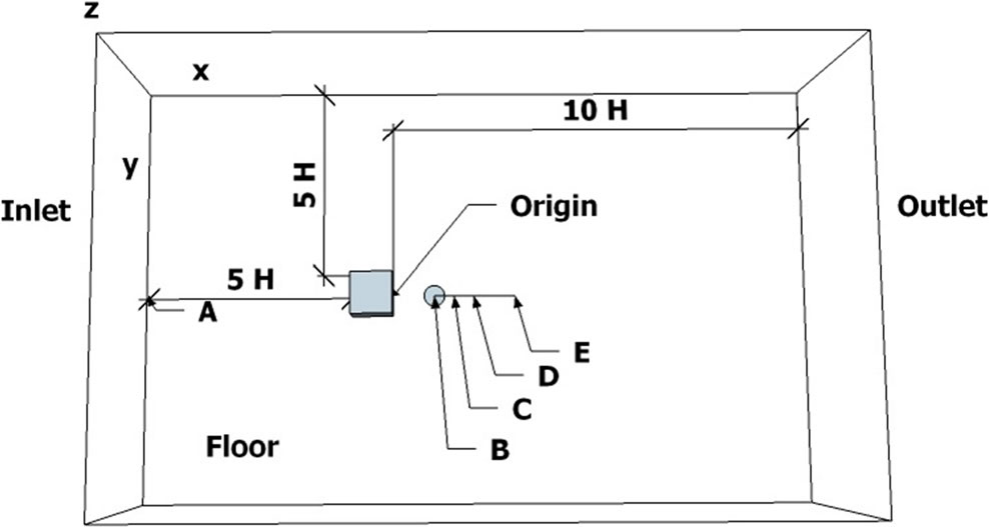
Computational domain and boundary conditions. The letters indicate the monitoring positions: A (*X*: −6H, *Y*:0, *Z*:0), B (H, 0, 0), C (1.5H, 0, 1.5H), D (2H, 0, 0), and E (3H, 0, 0)

The turbulent flow field around the SILSOE cube that is considered a standard test case for atmospheric flows around isolated buildings was selected also as a test case in the present work. Historically, the SILSOE cube experiment was performed at the SILSOE Research Institute, where a 6-m-high isolated cube was placed on a flat terrain in an open country site. Several different studies have been performed for analyzing the flow around this cube (Richards and Hoxey 1993; Richards and Hoxey 2006; Richards and Hoxey 2008; Richards and Hoxey 2012; Richards et al. 2001). Apart from the field experiments, wind tunnel and numerical studies have been also performed for the SILSOE cube (Hoxey et al. 2002; Richards and Norris 2015; Richards et al. 2002; Richards and Hoxey 1993; Richards and Norris 2011). It should be noted that the guidelines of the German Association of Engineers (VDI) recommend keeping the blockage effect below 10% (Franke et al. 2007). The present configuration and the selected monitoring locations are shown in Fig. 1, where the middle of the rear edge of the cube bottom face is selected as the origin of the coordinate system. The upstream boundary of the computational domain is 5H from the front face of the cubic building; the downstream computational boundary is 10H (Franke et al. 2007; Tominaga et al. 2008) from the rear cube face; the lateral width of the computational domain is 11H and its height is 5H (Zheng et al. 2020), where H is the height of the cube, resulting in the small estimated blockage effect value of 1.8%, which is smaller than the recommended value of 3% (Franke et al. 2007; Tominaga et al. 2008.

Since our focus is on the smoke dispersion and the determination of the toxic zones around the building due to different pool fire scenarios, the Reynolds number is kept constant at 4.1 × 10^6^, based on the free stream velocity and the height of the cube. Moreover, a wind orientation at 0^o^, parallel to the ground and normal to the front face of the cube, in the streamwise direction is considered. The center of the source emission, i.e., point B in Fig. 1, is located on the floor of the computational domain and at a distance H behind the cube. The two pool fires are simulated by a plume above a 3-m diameter pool of oil. Scenario 1 corresponds to a crude oil pool fire and Scenario 2 to a diesel pool fire. The fuel mass-loss $\overset{.}{m}$ and the total heat release rate $\overset{.}{\mathrm{q}}$(HRR) are calculated as in Babrauskas (1983) :

1 $$ \overset{.}{m}={\overset{.}{m}}_{\infty}\left(1-{e}^{- k\beta D}\right) $$

2 $$ \overset{.}{\overset{.}{q}=\overset{.}{m}\ \varDelta {H}_{c, eff}{A}_{pool}} $$

where ${\overset{.}{m}}_{\infty }$ is the infinite-diameter pool mass-loss rate, ΔΗ_*c*, *eff*_ is the heat of combustion, *A*_*pool*_ is the surface area of the pool, *β* is the mean beam length corrector, *k* is the absorption extinction coefficient of the flame, and *D* is the pool diameter.

Τhe crude oil and diesel pool fires have a total heat release rate $\overset{.}{q\ }$(HRR) of 7.8 MW and 13.5 MW, respectively. The convective part of the HRR for both scenarios is ${\overset{.}{q}}_c=0.7\overset{.}{q}$. The smoke yield is an important parameter that defines the ratio of the produced smoke mass to the consumed fuel mass (kg smoke/kg fuel). The smoke yield for crude oil is between 10% and 15%, according to Evans et al. (2001) and here is taken to be 12.5% (Argyropoulos et al. 2010). The diesel oil is composed of 75% saturated and 25% aromatic hydrocarbons. Walton et al. (1995) assumed that its smoke yield varies between 15% and 20% (Argyropoulos et al. 2010) and an average value of 17.5% is considered here. The composition of emissions of a petroleum hydrocarbon fire is water vapor, carbon dioxide (92%), carbon monoxide (3.2%), and PM (5%) (Stout and Wang 2018). The pool fires are modelled as a source of a thermal gas which is injected normally into the ground. Smoke with 0.032 kg/s and 0.053 kg/s is released for the crude oil and diesel pool fires, respectively.

### Governing equations

The governing equations employed for LES are obtained by filtering the time-dependent Navier-Stokes equations. For compressible flows, the density-weighted (Favre-averaging) filtering operator is defined as $\overset{\sim }{\phi }=\overline{\rho \phi}/\overline{\rho}$. The LES approach used here is formulated by filtering the continuity, the Navier-Stokes, and energy equations:

3 $$ \frac{\partial \overline{\rho}}{\partial t}+\frac{\partial }{\partial {x}_j}\left(\overline{\rho}\tilde{u}_{j}\right) $$

4 $$ \frac{\partial \left(\overline{\rho\ }\tilde{u}_{i}\right)}{\partial t}+\frac{\partial \left(\overline{\rho\ }\tilde{u}_{i}\tilde{u}_{j}+\overline{p}{\delta}_{ij}\right)}{\partial {x}_j}=\frac{\partial {\sigma}_{ij}}{\partial {x}_j}-\frac{\partial {\tau}_{ij}}{\partial {x}_j}+\rho g $$

5 $$ \frac{\partial }{\partial t}\left(\overline{\rho\ }\tilde{h}_{s}\right)+\frac{\partial \left(\overline{\rho\ }\tilde{u}_{i}\tilde{h}_{s}\right)}{\partial {x}_i}-\frac{\partial \overline{p}}{\partial t}-\tilde{u}_{j}\frac{\partial \overline{p}}{\partial {x}_i}=-\frac{\partial }{\partial {x}_j}\left[\rho \left(\overset{\sim }{u_i{h}_s}-\tilde{u}_{i}\tilde{h}_{s}\right)\right] $$

where $\overline{\rho}$, ${\overset{\sim }{u}}_{\mathrm{i}}$, $\overline{p}$, and ${\overset{\sim }{h}}_{\mathrm{s}}$ are the resolved scales of the variables of density, velocity in the *i*=1, 2, 3 directions, pressure, enthalpy, *t* is the time, *g* is the acceleration of gravity, ${\sigma}_{\mathrm{i}\mathrm{j}}={\mu}_{\mathrm{lam}}\Big(\left[\frac{\partial {\overset{\sim }{\mathrm{u}}}_{\mathrm{i}}}{\partial {\mathrm{x}}_{\mathrm{j}}}+\frac{\partial {\overset{\sim }{\mathrm{u}}}_{\mathrm{j}}}{\partial {\mathrm{x}}_{\mathrm{i}}}-\frac{2}{3}{\updelta}_{\mathrm{i}\mathrm{j}}\frac{\partial {\overset{\sim }{\mathrm{u}}}_{\mathrm{k}}}{\partial {\mathrm{x}}_{\mathrm{k}}}\right]$ is the deviatoric Newtonian stress tensor, *μ*_lam_ is the dynamic viscosity, *τ*_ij_ is the (SGS) stress tensor and *Q*_j_ is the heat transport flux.

The compressible form of the sub-grid stress tensor is defined as:

6 $$ {\tau}_{ij}=\overline{\rho}\overset{\sim }{u_i{u}_j}-\overline{\rho}\tilde{u}_{i}\tilde{u}_{j} $$

This term is split into its isotropic and deviatoric parts. The deviatoric part is modelled with the compressible form of the Smagorinsky model:

7 $$ {\tau}_{ij}-\frac{1}{3}{\tau}_{kk}{\delta}_{ij}=-2{\mu}_t\left({\overset{\sim }{S}}_{ij}-\frac{1}{3}{S}_{kk}{\delta}_{ij}\right) $$

The isotropic part ($\frac{1}{3}{\tau}_{kk}{\delta}_{ij}$) of the sub-grid scale stresses *τ*_*kk*_ is not modelled but is added at the filtered static pressure term. The rate-of-strain tensor for the resolved scale, ${\overset{\sim }{S}}_{ij}$, is defined as ${\overset{`}{S}}_{ij}\equiv \frac{1}{2}\left(\frac{\partial {\overset{`}{u}}_i}{\partial {x}_j}+\frac{\partial {\overset{`}{u}}_j}{\partial {x}_i}\right)$ and the eddy viscosity *μ*_*t*_ is modelled by ${\mu}_t=\overline{\rho\ }{L}_s^2\left|{\overset{\sim }{S}}_{ij}\right|$ where *L*_s_ is the mixing length for sub-grid scales L_s_ = min(κd, C_s_Δ), with *κ*=0.41 the von Kármán constant, *d* the distance to the closest wall, *C*_s_ kept constant at 0.17, and *Δ* = ∀^1/3^ the local grid scale computed according to the volume of the computational cell.

The sub-grid enthalpy flux term is approximated using the gradient:

8 $$ \overline{\rho}\left(\overset{\sim }{u_i{h}_s}-\tilde{u}_{i}\tilde{h}_{s}\right)=-\frac{\mu_{SGS}{C}_p}{{\mathit{\Pr}}_{SGS}}\frac{\partial \overset{\sim }{T}}{\partial {x}_j} $$

where *T* is the temperature, *C*_*p*_ is the specific heat, and *Pr*_*SGS*_ is the sub-grid Prandtl number that is kept equal to 0.85.

The filtered species transport equation of the smoke concentration is expressed by:

9 $$ \frac{\partial }{\partial t}\left(\overline{\rho}\overset{\sim }{c}\right)+\frac{\partial }{\partial {x}_i}\left(\overline{\rho}\overset{\sim }{c}\ \tilde{u}_{i}\right)=\frac{\partial }{\partial {x}_j}\left({J}_i\right)+S $$

where *c* is the smoke concentration, and *S* includes all source terms inside the flow field.

The total diffusion flux of species due to molecular and turbulence diffusion is expressed as:

10 $$ {J}_i=-\left(\overline{\rho}{\overset{\sim }{D}}_{i,m}+\frac{\mu_t}{Sc_t}\right)\frac{\partial \overset{\sim }{c}}{\partial {x}_j} $$

where ${\overset{\sim }{D}}_{i,m}$is the diffusion coefficient for the species in the mixture and *Sc*_*t*_ is the turbulent Schmidt number, which is kept constant at 0.7 here and may be varied between 0.2 and 1.3 (Tominaga and Stathopoulos 2007).

The non-dimensional concentration coefficient, *K*, is a measure of the mean concentration according to Li and Meroney (1983) and Saathof et al. (1995), defined by:

11 $$ K=\frac{\left({C}_{measured}/{C}_{source}\right){U}_H\ {H}^2}{Q_{source}} $$

where *C*_*measured*_ is the tracer concentration, *C*_*source*_ is the tracer concentration at the source, *Q*_*source*_ is its release rate, and *U*_*H*_ is the velocity at the building height.

In a fire accident, smoke buoyant plumes move upward and influence its concentration and dispersion. At the same time, the flow of wind bends the fire plume and traps the fire pollutants inside the cube recirculation zone. The ratio of the thermal buoyancy forces to the wind convection forces is described by the Richardson number as:

12 $$ Ri=\frac{gH\varDelta T}{T_{\infty }{U}_H^2} $$

where Δ*T* is the temperature difference between the fire plume and the air temperature *T*_∞_.

At low Richardson numbers, the temperature difference, Δ*T*, is small and the buoyancy force is a second-order effect. In the present scenarios, it is found that the Richardson number takes the values 2.36 and 2.56 for the crude oil and the diesel pool fires, respectively. These quite high Richardson numbers indicate that the buoyancy force is important in driving the smoke and mixing the smoke products.

### Boundary conditions

The velocity distribution of the atmospheric boundary layer at the inlet is experimentally defined by Richards and Hoxey (1993) as:

13 $$ U(z)=\frac{u_{\ast }}{\kappa}\mathit{\ln}\left(\frac{z+{\mathrm{z}}_0}{{\mathrm{z}}_0}\right) $$

14 $$ {u}_{\ast }=\frac{\kappa {U}_{ref}}{\mathit{\ln}\left(\frac{z_{ref}}{{\mathrm{z}}_0}\right)} $$

where the wind speed at the reference height *z*_ref_ = 10 m is *U*_ref_ = 10.13 m/s and the roughness height is *z*_0_ = 0.01 m. For more detailed information for the SILSOE’s cube atmospheric boundary layer, several references exist (Hoxey et al. 2002; Richards and Norris 2015; Richards and Hoxey 2012). This study is limited to a wind flow direction normal to the front face of the cube (0° angle), with a vortex topology described by Martinuzzi and Tropea (1993). In the case of a wind at 45° angle, strong vortices are generated from the swept-back leading edges and a stronger downwash in the wake is changing the vortex topology (Castro and Robins 1977).

The inlet velocity profile is indicative of an atmospheric boundary layer of near-neutral stability (Richards et al. 2001). Some studies consider the effect of atmospheric stability on the flow field (Pieterse and Harms 2013) and its effect on the near-field pollutant dispersion from rooftop pollutant emissions (Jeong and Kim 2018). The atmospheric stability could affect the main airflow characteristics around the cube such as the reattachment length on the roof cavity, the horizontal cavity length and width near the surface, and the spread of the plume on the leeward surface (Jeong and Kim 2018).

The turbulent kinetic energy, *k*(*z*), and turbulent dissipation rate, *ε*(*z*), at the inlet are defined as:

15 $$ k(z)=\frac{u_{\tau}^2}{\sqrt{C_{\mu }}} $$

16 $$ \varepsilon (z)=\frac{u_{\tau}^3}{\kappa\ \left(z+{z}_0\right)} $$

where *C*_μ_ = 0.09 is the model’s constant, *u*_τ_ = 0.63 m/s the friction velocity, and *κ*=0.4 the von Karman constant.

The mean velocity and pressure fields are initialized from a standard *k*- *ε* simulation, and then the vortex method (ANSYS Inc. (US) 2016) is implemented to help in establishing realistic turbulence profiles as well as reduction of the initial transient time before statistical stationarity is reached, using the well-known strategy of Vasaturo et al. (2018).

**Fig. 2:**
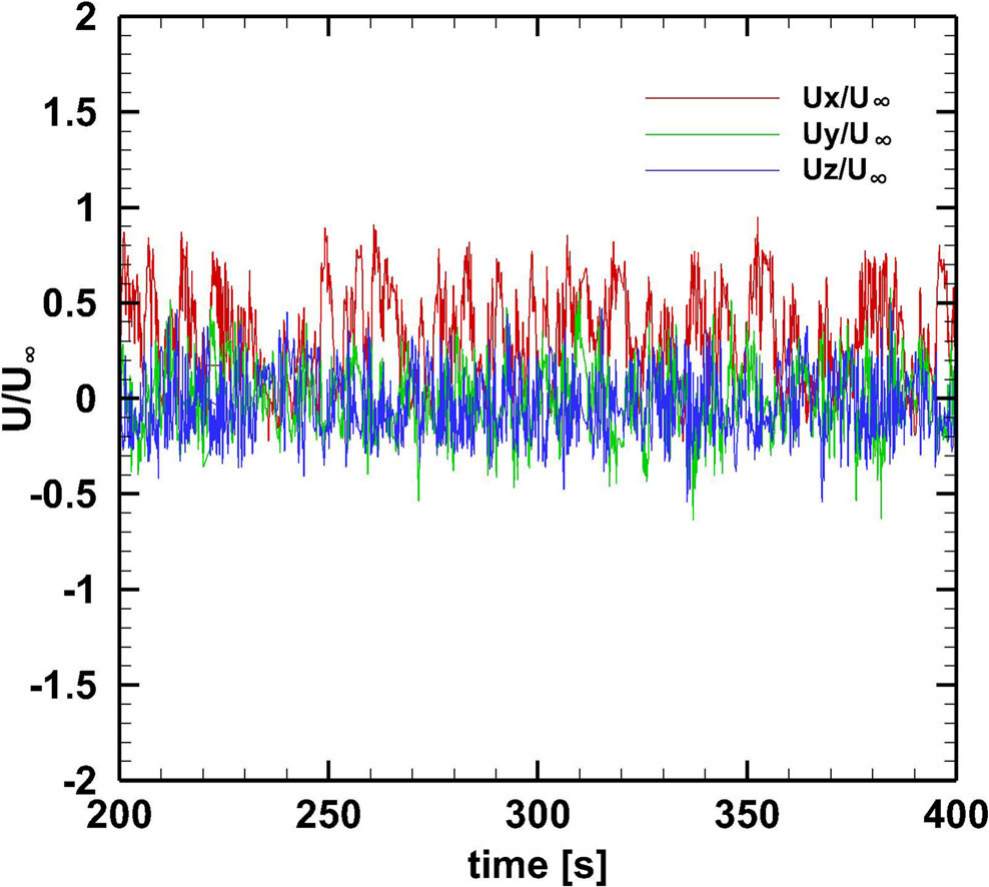
Instantaneous velocity fluctuations of (***u***_**x**_/***U***_**∞**_**,*u***_**y**_/***U***_**∞**_**,*u***_**z**_/***U***_**∞**_) at points *X*: 0.08H, *Y*: 0, *Z*: H during the time period of 300 to 400 s

For the first 2.5 s of the transient simulation, a small-time step of Δ*t*=0.0005 s and 100 sub-iterations per iteration are applied. For the next 200 s of the transient simulation, a smaller time step (Δ*t*=0.005 s) and 50 sub-iterations per iteration are applied until a stationary state is reached. Figure 2 shows the monitoring of local quantities (*u*_x_/*U*_∞_, *u*_y_/*U*_∞_, *u*_z_/*U*_∞_) at points *X*: 0.08H, *Y*: 0, and *Z*: H with stationary conditions and within the time period from 200 to 400 s. For this time period, the mean flow values are computed. The non-dimensional mean values of *u*_x_/*U*_∞_ at this point are for the time period 25 to 100 s 0.2919, 100 to 175 s 0.3166, 175 to 275 s 0.3236, 275 to 375 s 0.3319, and 375 to 420 s 0.3319.

It should be noted that a second approach is to evaluate the targeted velocity and turbulence intensity profiles at the building location (Guichard 2019). At the lateral sides of the domain, periodic boundary conditions are applied. Well-established conditions at the outlet, where all derivatives vanish, and the pressure is kept equal to zero, are used.

### Numerical details

The CFD software package Ansys Fluent 17 is used for the simulations of this work as it has been used on related studies by Guichard (2019) for the SILSOE cube, by Chew et al. (2018) for the buoyant flows in street canyons, and by Idrissi et al. (2019) for the air pollutant dispersion in complex urban areas. The nonlinear terms are discretized with a second-order upwind scheme. A second-order scheme is used also for the discretization of all other terms of Eqs. (6) to (19), combined with the PISO scheme for the pressure and velocity coupling. The convergence criteria are kept below the value of 10^−4^, based on the absolute error for all quantities. A time step of 0.005 s is used in all simulations with implicit time stepping which resulted in a cell convective Courant number with a minimum value of 0.0016, a maximum value of 0.929, and an average value of about 0.0167. In order to initialize the flow field, a steady-state simulation was performed with the application of the standard *k*-epsilon Reynolds averaged model.

The grid mesh is important in urban aerodynamic simulations of complex terrains where the flow characteristics determine the required grid resolution. Effort is made to reduce the cost of the simulations, while maintaining accurate results. Τhe choices on the type of mesh and its resolution have a significant effect on the accuracy of the results for the flow around buildings and special attention should always be paid (Hefny and Ooka 2009). Structured and unstructured grids could be used in studying the airflow in urban environments, with the structured grids requiring less computing time and leading to stable computational solution. As tetrahedral volume meshes could lead to numerical errors (Hefny and Ooka 2009), a fine enough grid is adopted around the cube in order to assure the quality of the results.

For the near-wall cells, body-fitted prismatic or hexagonal cells have to be used (Casey and Wintergerste 2000).

**Fig. 3:**
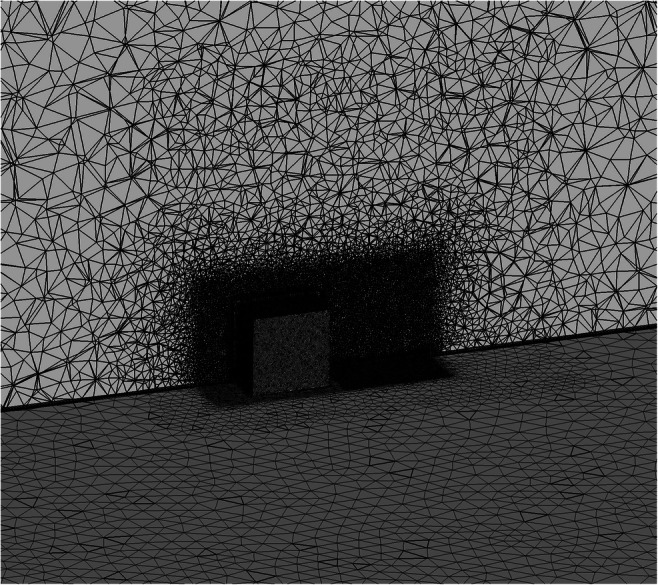
Mesh arrangement at the symmetry plane of the computational domain

The flow domain is clustered into several sub-domains near the cube, each with a suitable grid mesh to ensure high accuracy and optimal prediction of the flow features. A hybrid mesh is used here, with an unstructured tetrahedral grid outside the boundary layer and a prismatic mesh inside the boundary layer (Fig. 3). The near-wall mesh is fine enough to resolve the laminar sub-layer, with the normal distance from the wall of the first cell to be less than 7.5x10^−5^ m. In order to estimate the error associated with the grid sensitivity, the grid convergence index (GCI) method is used (Chatzimichailidis et al. 2019; Hefny and Ooka 2009; Roache 1997).

Three different grids of successively increasing resolution are used, while the error is estimated from two different grids using the formula:

17 $$ GSI=\frac{f_2-{f}_1}{1-{r}^p} $$

where *f*_2_ is the numerical solution obtained by a coarse grid, *f*_1_ is the numerical solution obtained by a finer grid, *r* is the refined factor between the coarse and the finer grid, and *p* is the accuracy of the algorithm (*p* = 2, here). Grids consisting of 1,527,575, 2,415,662, and 3,659,771 mesh points in the coarse, medium, and fine scenarios, respectively, are used. Thus, the refine factor between two successive grids is about *r* = 1.5.

**Fig. 4:**
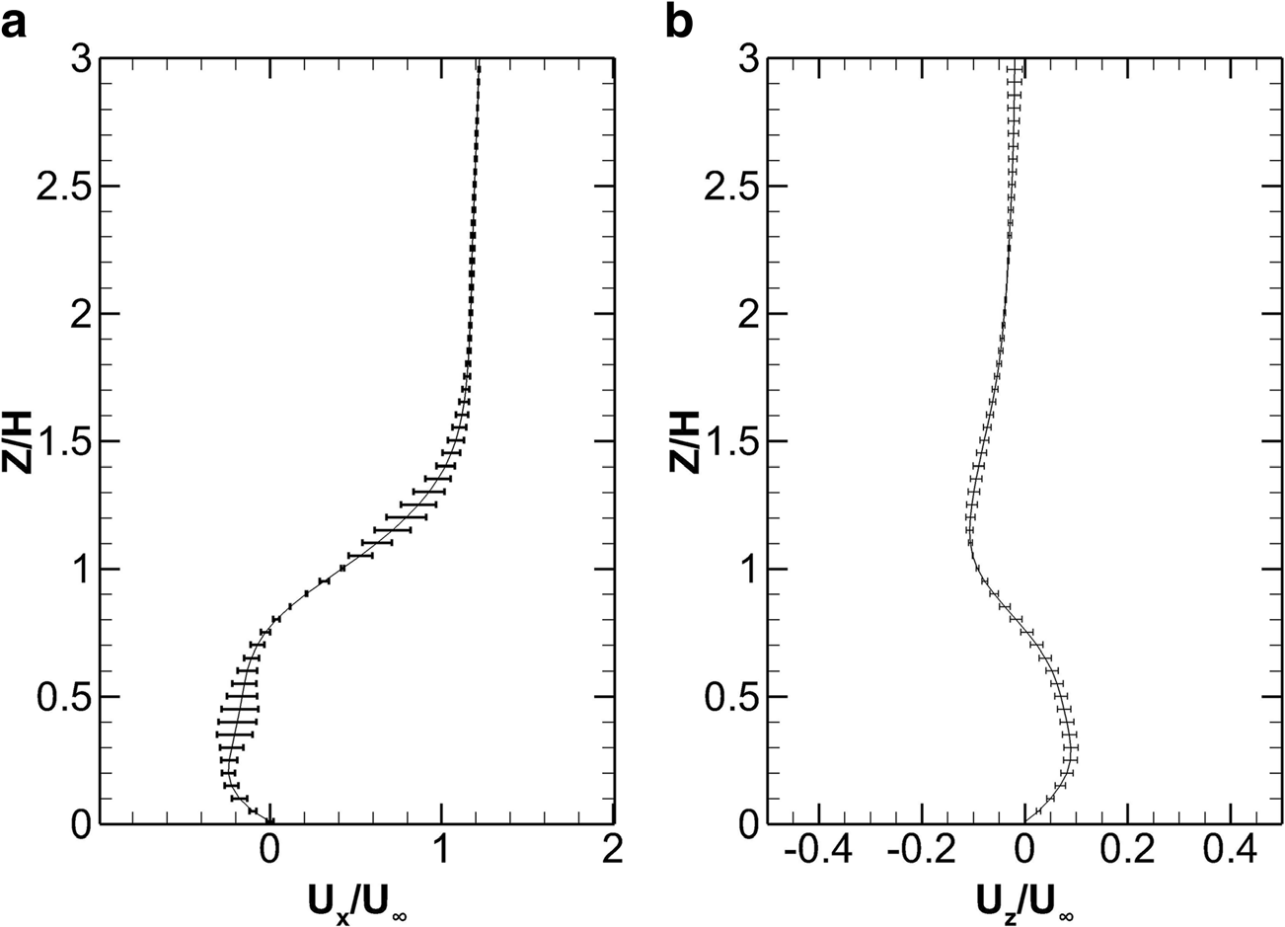
The GCI error bars estimated from the fine and medium grids for the mean velocities *U*_x_/*U*_∞_ and *U*_*z*_/*U*_∞_, based on the medium grid, at position 0.5H behind the cube

Figure 4 shows the GCI error bars in the streamwise position of 0.5H behind the cube, inside the recirculation zone, and the average values are found to be 2.56%, 0.93%, and 1.37%, for *U*_x_/*U*_∞_, *U*_*z*_/*U*_∞_ and *U*_*y*_/*U*_∞_, respectively, based on the coarse and the medium grids and 0.34%, 0.63%, and 0.36%, respectively, when using the medium and the fine grids. Thus, going from the medium to the fine grid, the estimated errors are about an order of magnitude smaller and therefore for the present simulations the medium grid is selected.

## Results and discussion

### Model validation

**Fig. 5:**
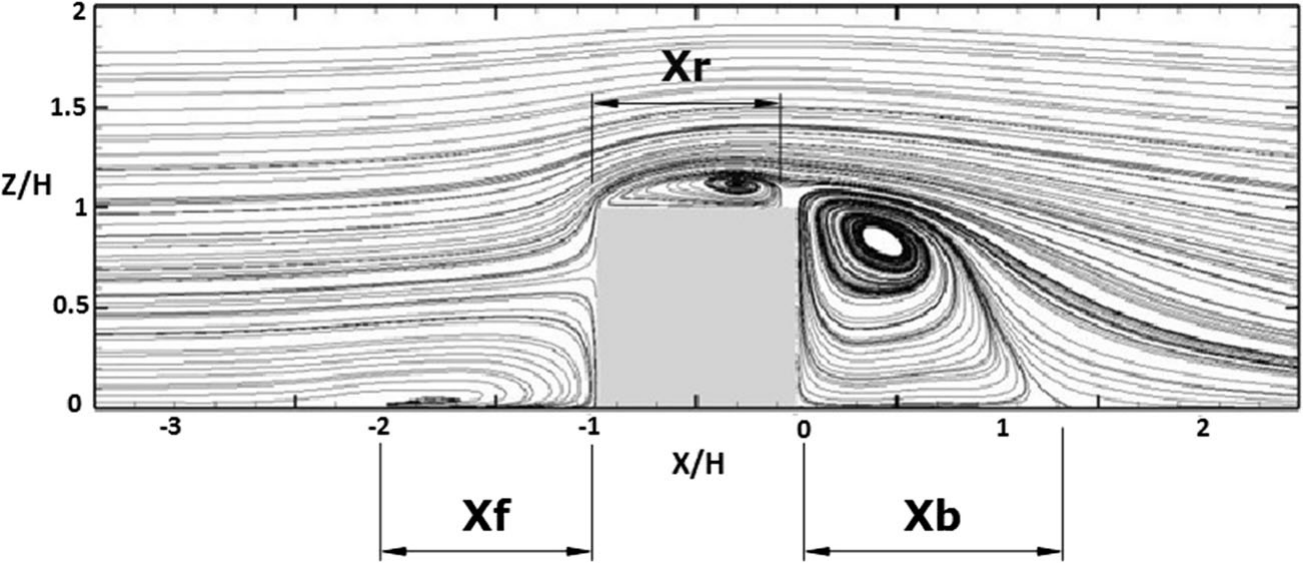
Streamlines of the mean flow on the symmetry plane, for present results, and the characteristic separation lengths

Initially, the flow around a cubical building without smoke dispersion is simulated. The results are compared against the full-scale SILSOE cube experiments of Hoxey et al. (2002), the improved delayed detached eddy simulation (IDDES) results of Hu et al. (2018), and the LES results of Richards and Norris (2015). The comparison is based on the flow patterns and pressure coefficient distribution around the cube. The mean flow field in the form of streamlines at the symmetry plane is shown in Fig. 5, showing flow separation on the roof, and recirculation zones windward and downstream of the cube. The lengths of the main separation regions upstream, downstream of the cube, and on the roof are denoted by *X*_*f*_, *X*_*b*_, and *X*_*r*_, respectively, and compared in Table 1.

**Table 1:** Lengths of main separation regions

	Scenario	Upstream separation *X*_*f*_	Roof reattachment *X*_*r*_	Downstream reattachment *X*_*b*_
Hoxey et al. (2002)	Full-scale experiment	0.75	0.57	1.4
Richards and Norris (2015)	LES	0.9	0.9	~1.4
Hu et al. (2018)	IDDES	Not mentioned	0.94	1.31
Present results	LES	0.99	0.9	1.37

As the flow approaches the leeward surface of the cube from the roof, the main separation vortex appears. In the front corner of the cube with the ground and in the corresponding side corners, a horseshoe vortex (Theodorsen 1952) is formed which has its head in the front corner (forming a recirculation zone there) and its legs in the corresponding side corners, binding the cube. The upstream recirculation zone is found experimentally to extend to *X*_*f*_ = 0.75 *H* (Hoxey et al. 2002). The present study overestimates this zone which is found to be *X*_*f*_ = 0.99 *H*, in good agreement with the LES results of Richards and Norris (2015). Moreover, the computed length of the separation zone on the roof of the cube, *X*_*r*_ = 0.9 H, is found to be equal to that predicted by Richards and Norris (2015) and Hu et al. (2018), while the flow patterns look identical. The predicted reattachment length is found to be higher than the measured value of *X*_*r*_ = 0.57 H. The recirculation zone downstream of the cube is similar for all the different scenarios studied with an approximate value of the reattachment length *X*_b_ ≈ 1.4H. The flow field behind the cube could be separated into two different zones: the cavity zone with low velocities and high turbulence (Huber 1981) and the near-wake zone after the cavity.

**Fig. 6:**
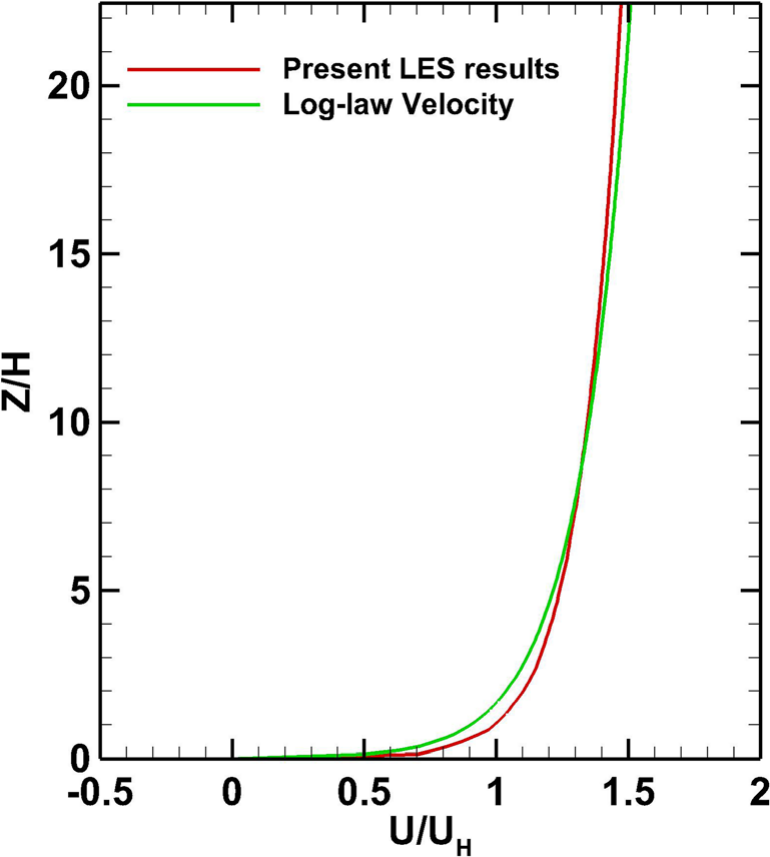
Inlet mean velocity distribution normalized with the velocity at the cube height

The velocity profile at the inlet A is illustrated in Fig. 6 and is in good agreement with the log-law velocity profile of Eq. (13). In order to validate the flow behavior around the cube, the pressure coefficient is calculated as:

18 $$ {C}_p=\frac{p-{p}_{ref}}{\frac{1}{2}{\rho}_{ref}{U}_{\infty}^2} $$

where *p* is the fluid static pressure, and *p*_*ref*_, *U*_∞_, and *ρ*_*ref*_ the free stream static pressure, velocity, and density, respectively.

**Fig. 7:**
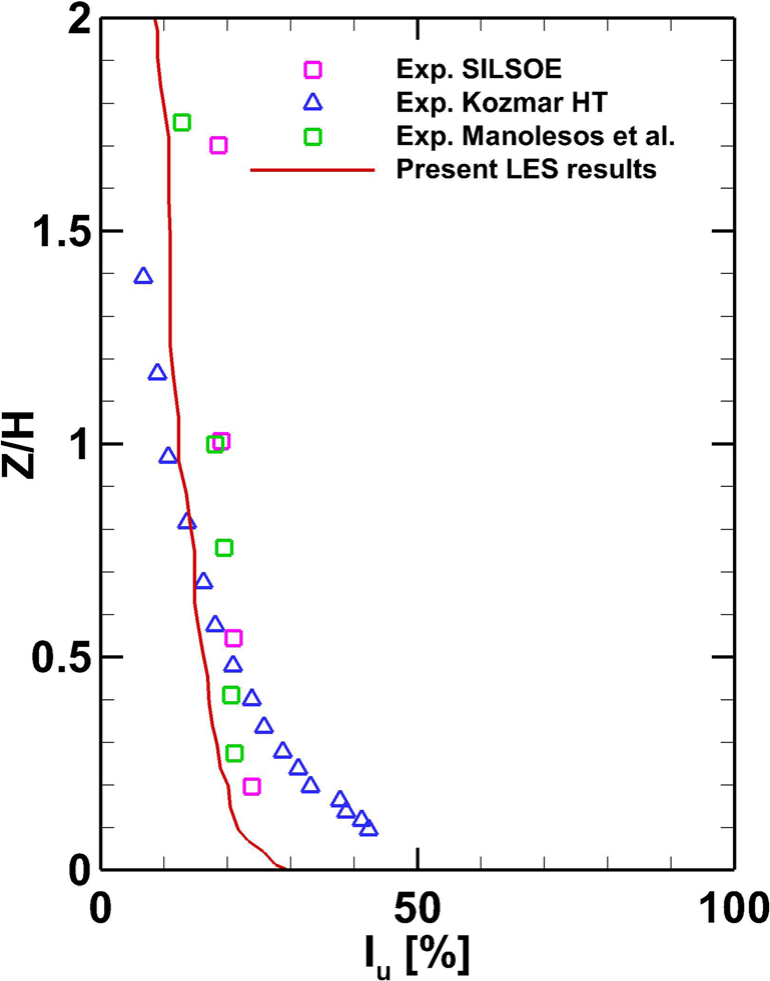
Mean streamwise turbulence intensity at the inlet of the computational domain.

Figure 7 shows the vertical profile of the streamwise turbulence intensity from the present LES in comparison with the experimental data from SILSOE and other studies (Kozmar 2020; Manolesos et al. 2018; Richards et al. 2007). This comparison indicates good agreement with the SILSOE’s and Manolesos experimental data.

**Fig. 8:**
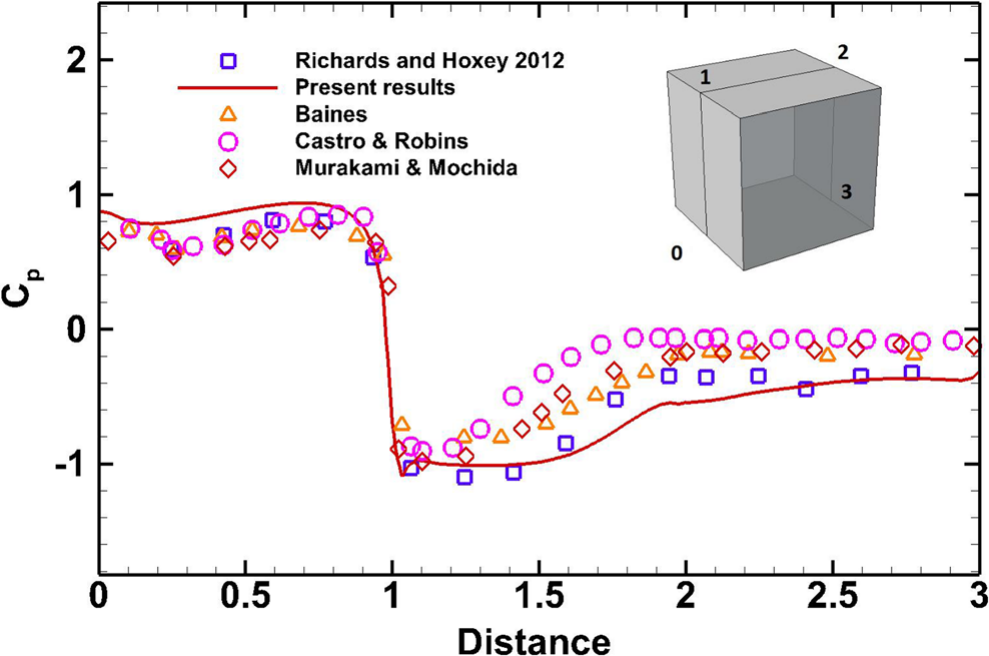
Pressure coefficient distribution along the symmetry plane of the cube.

The pressure coefficient profile around the SILSOE cube is illustrated in Fig. 8, and it is found to be in good agreement with the measurements of Richards and Hoxey (2012) and have similar characteristics with other experiments obtained in wind tunnels with turbulent boundary layers (Baines 1963; Castro and Robins 1977; Murakami and Mochida 1988).

**Fig. 9:**
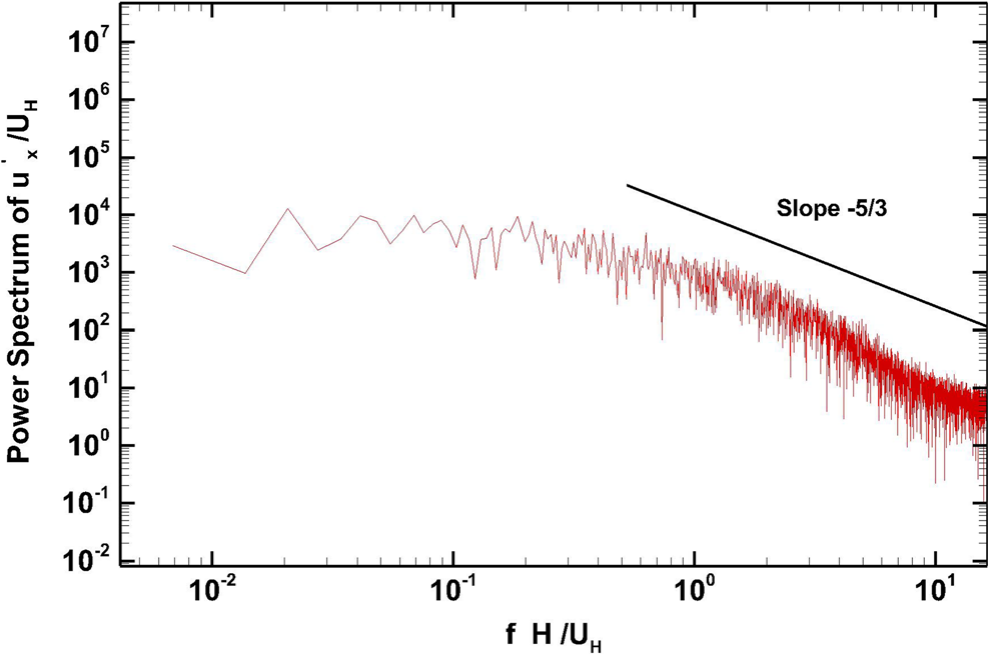
Normalized power spectra of streamwise velocity fluctuations at position C (1.5H, 0, 1.5H)

Figure 9 shows the non-dimensional power spectrum of the streamwise turbulent velocity fluctuation at position C (1.5H, 0, 1.5H) where the −5/3 Kolmogorov law (in non-weighted representation) is verified.

**Fig. 10:**
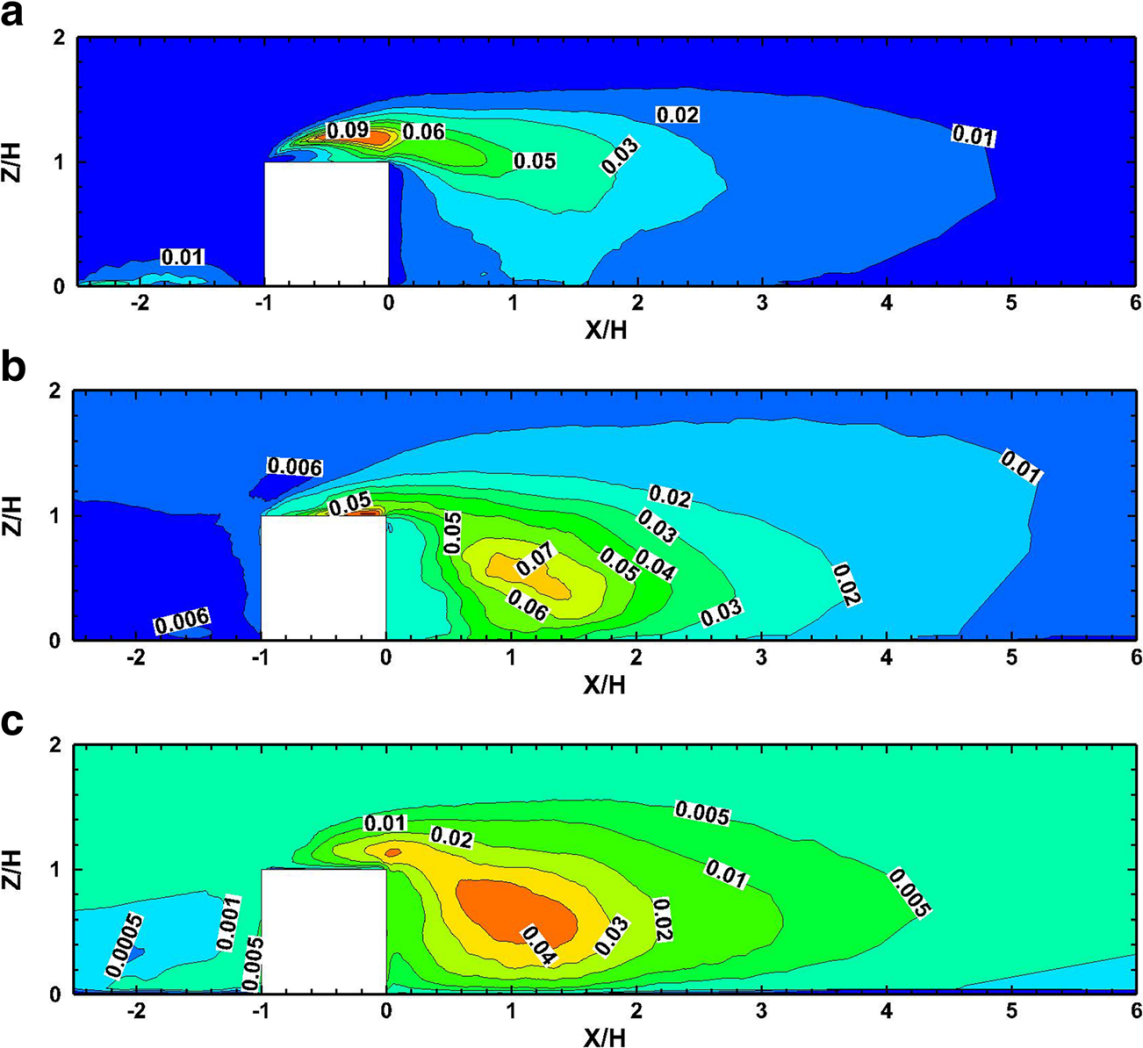
Mean-square intensities at the symmetry plane of the a streamwise velocity$\kern0.5em \overline{{{\boldsymbol{u}}_{\mathbf{x}}^{\prime}}^{\mathbf{2}}}$, b transverse velocity $\overline{{{\boldsymbol{u}}_{\mathbf{z}}^{\prime}}^{\mathbf{2}}}$, and c and spanwise velocity $\overline{{{\boldsymbol{u}}_{\mathbf{y}}^{\prime}}^{\mathbf{2}}}$ components normalized by ${U}_{\infty}^2$

Figure 10 presents the contours of the mean-square turbulent stresses at the symmetry plane. The turbulence streamwise mean-square intensities $\overline{{u_{\mathrm{x}}^{\prime}}^2}$ have their maximum values above the cube roof where the shear layer separates and then reattaches as is shown in Fig. 10a. Large values exist also near the ground at the upwind area of the cube where the horseshoe vortex is formed. Moreover, the transverse mean-square intensities $\overline{{u_{\mathrm{z}}^{\prime}}^2}$ have maximum values at the trailing edge of the cube, where the shear layer formed on the cube roof interacts with the recirculating flow behind it, as it is shown in Fig. 10b. In addition to the local maximum of the transverse mean-square intensity at the trailing edge of the cube, another maximum occurs at the position *X*/H = 1.6, *Y*/H = 0.54. In this region, enhanced mixing occurs. Finally, Fig. 10c shows the spanwise mean-square intensities $\overline{{u_{\mathrm{y}}^{\prime}}^2}$ to have similar characteristics as the transverse ones. All mean-square intensities show high values in the horseshoe vortex region near the upwind wall of the cube. The present LES mean-square intensities of the three velocity components at the symmetry plane, indicated in Fig. 10, show the same behavior with the corresponding intensities based on the DNS numerical data of Yakhot et al. (2006).

### Hazardous material dispersion

A fire source in the near wake of the cube produces large wind fluctuations and non-uniform concentration distributions of smoke and gases. The pool fire is considered here as a local heat and chemical species source that is affecting the density of the air. Even though the produced thermal plume causes strong buoyant forces, its effect on the size of the cavity zone is small due to its locality. The size of the recirculation zone for the two case studies has been changed comparatively very little. The recirculation zone is found to be *X*_*b*_ = 1.31 *H* for the crude oil pool fire and *X*_*b*_ = 1.285 *H* for the diesel pool fire. The buoyant forces cause a small decrease in the recirculation zone and an increase in the height of the cavity zone. These results are in agreement with the studies of Olvera et al. (2008) and Smith et al. (2001). Brzoska et al. (1997) similarly observed that when the plume extent is within the recirculation zone, its size is not affected drastically, and is independent of the source location.

**Fig. 11:**
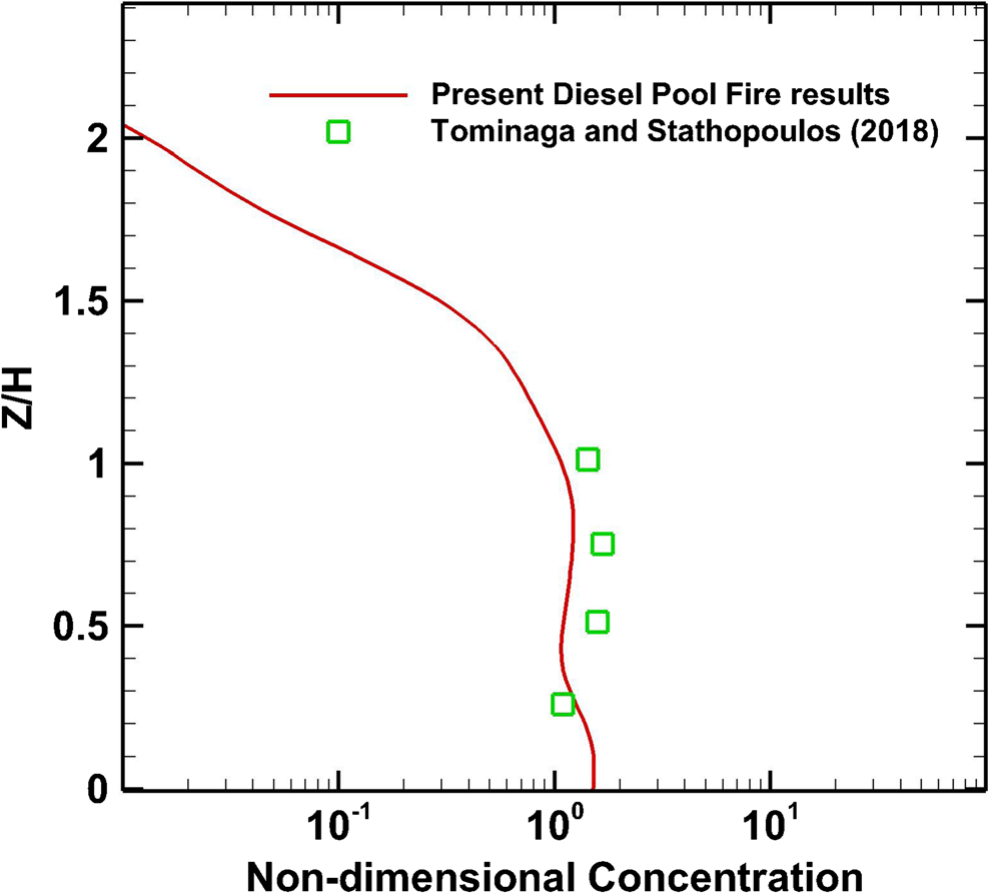
Mean smoke concentration profile behind the building at *X*/H = 2, position D

The present numerical results are validated against the experimental data of Tominaga and Stathopoulos (2018), which were obtained in the wind tunnel of the Institute of Industrial Science at the University of Tokyo. This wind tunnel experiment employs mixtures of different gases such as C_2_H_4_ (neutral), He and C_2_H_4_ (light), and SF_6_ and C_2_H_4_ (heavy gases) for the study of the dispersion around a cubical obstacle. The mixture release point is located at position *X*/H=0.5, behind the cube and within the recirculation zone. The light gas is considered to play the role of a thermal plume. In this work, the computational results for smoke dispersion of the diesel pool fire are compared with the aforementioned experimental data. As shown in Fig. 11, the numerical results of the concentration of the diesel pool fire accident match well with the dispersion of the light gas experimental data at position D (*X*/H=2). As described before, the flow field is stationary, but at the moment the fire was initiated, the stationarity temporarily was broken. The monitoring of the mean non-dimensional concentration at the same point (*X*: 0.08H, *Y*: 0, *Z*: H) shows the following values: 6.34 ∙ 10^−5^ for the time period 100–200 s, 7.234 ∙ 10^−5^ for 200–300 s, and 7.693 ∙ 10^−5^ for 300–400 s. This period is considered as the early detection time before mitigation actions can take place.

For the next 200 s, the smoke dispersion reaches the wake zone limits, and for the time between *t* = 200 to 400s, averaging of the mean concentration quantities is applied in order to define the toxic zones for an early accident detection and its mitigation. The mean values of the flow are calculated for a transient flow with time step of Δ*t*=0.005 s and 10 iterations per time step.

**Fig. 12:**
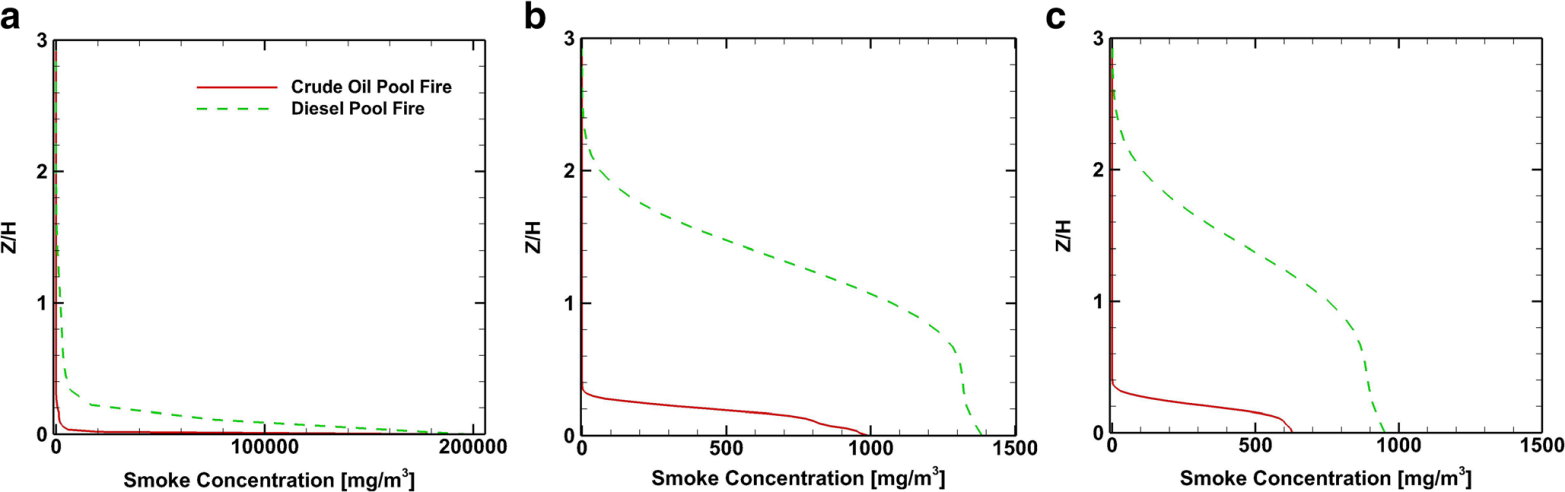
Time average smoke concentration coefficient profiles at positions *X*/H=1, 2, and 3. **a** Position B - *X*/H=1. **b** Position D - *X*/H=2. **c** Position E - *X*/H=3

**Fig. 13:**
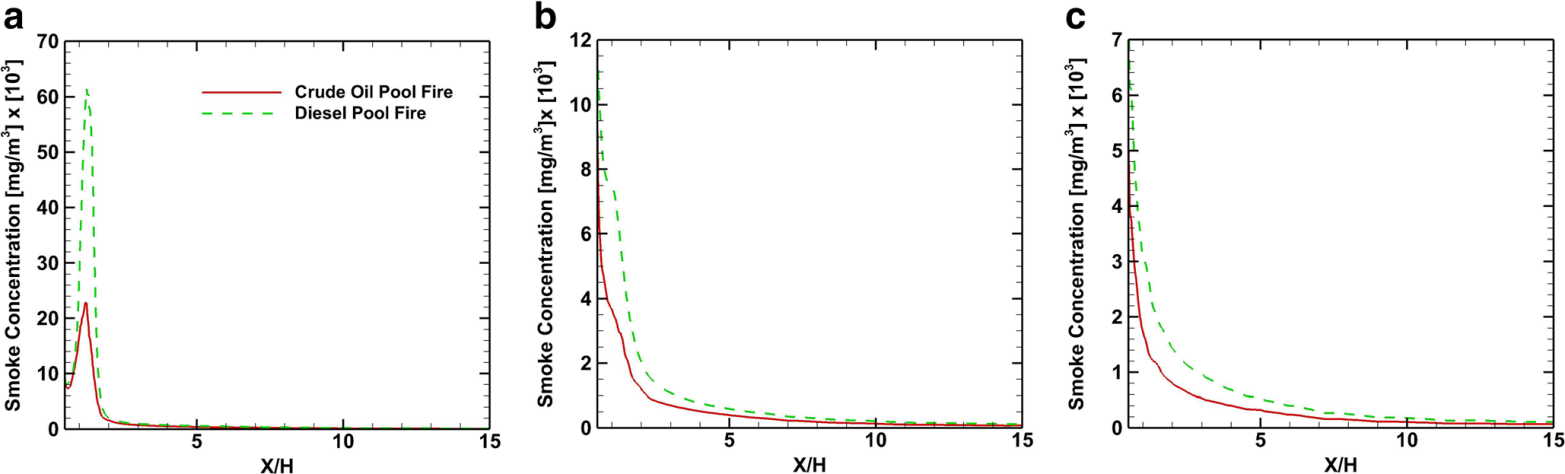
Time average smoke concentration coefficient profiles at *Z*/H=0.33, 0.5, and 1. **a** *Z*/H=0.33. **b** *Z*/H=0.5m. **c** *Z*/H=1

Figure 12 shows the smoke concentration at positions B, D, and E at *X*/H=1, 2, and 3, respectively, for the two scenarios studied. The smoke concentration just above the source (point B) has a similar distribution for both accidents. At positions D and E, the smoke concentration distributions show important differences between crude and diesel oil scenarios. In both scenarios, the plume spreads along the wall-normal direction away from the source. At the fire position B and at height Z/H=0.2, the smoke concentration for the diesel is 9 times higher than that of the crude oil fire. For the same height at position D, the product concentration from the diesel is 1.72 times higher than of the crude oil fire, while at point E is 1.56 times higher. These differences in the smoke mean concentration are primarily because the diesel pool fire produces higher amounts of smoke and secondary to the turbulence dispersion of the pollutant smoke. Figure 13 shows the profiles of mean smoke concentration at heights *Z*/H=0.33, 0.5, and 1.

The mechanism of smoke dispersion inside the wake zone for the convective and turbulent concentration fluxes is examined using the LES results. The definition of the time average filtered convective flux in the *x*-direction may be defined as ${q}_{\mathrm{x},\mathrm{convective}}=<{\overset{\sim }{u}}_{\mathrm{x}}><\overset{\sim }{c}>$ and for the *z*-direction as ${q}_{\mathrm{z},\mathrm{convective}}=<{\overset{\sim }{u}}_{\mathrm{z}}><\overset{\sim }{c}>$. Similarly, the definition of the time average turbulent flux in the *x*-direction may be defined as ${q}_{\mathrm{x},\mathrm{turbulent}}=<\overset{\sim }{u_{\mathrm{x}}^{\prime }}\overset{\sim }{c^{\prime }}$> and for the *z*-direction as ${q}_{\mathrm{z},\mathrm{turbulent}}=<\overset{\sim }{u_{\mathrm{z}}^{\prime }}\overset{\sim }{c^{\prime }}>$, where $\overset{\sim }{u_{\mathrm{x}}^{\prime }}$, $\overset{\sim }{u_{\mathrm{z}}^{\prime }}$, and $\overset{\sim }{c^{\prime }}$ are the resolved fluctuations (Gousseau et al. 2011). In order to express the above quantities in non-dimensional form, the reference flux concentration is defined:

19 $$ {q}_0={C}_0{U}_H $$

and the reference concentration is given as:

20 $$ {C}_0=\frac{Q_e}{H^2{U}_H} $$

where *Q*_e_ is the pollutant release rate.

**Fig. 14:**
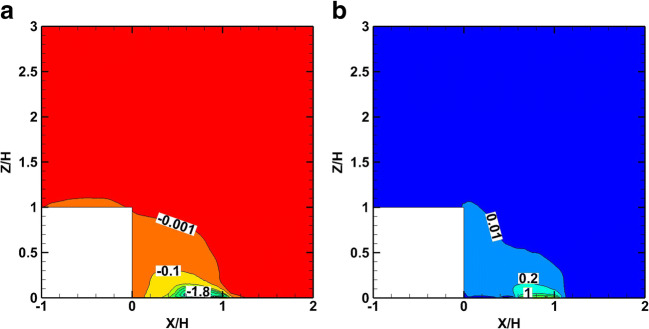
Distribution of time-averaged convective smoke mass fluxes in the cube symmetry plane for the crude oil fire. a ***q***_**x**, **conv**_/***q***_**0**_. b ***q***_**z**, **conv**_/***q***_**0**_

**Fig. 15:**
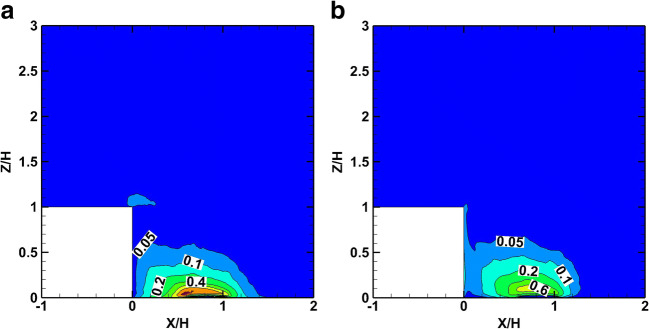
Distribution of time-averaged turbulent mass fluxes on the symmetry plane for the crude oil fire. **a** ***q***_**x**, **turb**_/***q***_**0**_. **b** ***q***_**z**, **turb**_/***q***_**0**_

The non-dimensional convective smoke mass flux of the crude oil plume fire in the *x*-direction (*q*_x, conv_/*q*_0_) is shown in Fig. 14a, and for the *z*-direction (*q*_z, conv_/*q*_0_) in Fig. 14b. Similarly, the turbulent mass flux in the *x*-direction (*q*_x, turb_/*q*_0_) is shown in Fig. 15a and for the *z-*direction (*q*_z, turb_/*q*_0_) is shown in Fig. 15b. Because of the similarity between the crude oil fire and the diesel pool fire non-dimensional results, only the crude oil fire results are presented.

As shown in Fig. 14a, the convective mass flux, *q*_x, conv_,is mainly towards the leeward face of the cube at the symmetry plane due to the negative velocity created into the cavity zone behind the cube. At the same plane, the vertical velocity of the smoke plume creates a positive convective mass flux, *q*_z, conv_,which is limited inside the recirculation zone behind the cube.

As shown in Fig. 15, the turbulent mass fluxes are significant in the wake zone and influence the smoke dispersion. The turbulent mass fluxes operate as a diffusion mechanism directed from the high towards the low concentration mass values. An amount of smoke is trapped inside the cavity due to the air recirculation. The negative *x*-velocities inside the cavity zone transport the plume towards the leeward face of the cube in both scenarios. The magnitude of the convective mass is dominant except for the areas where the velocity is very low.

### Toxic zones

The knowledge of hazardous zones can provide support for optimizing emergency plans against toxic gas leakage and dispersion accidents (Yang et al. 2020). The risk zone in a scenario of fire is an area of high heat flux and toxic substances. The risk zone is usually a semi-spherical area which has as center the source emission and extends to the limits where safety conditions exist. In order to determine the risk zones, the smoke level concentration has to be specified. Therefore, various zones are defined (Assael and Kakosimos 2010): the LC50 is a zone where a possibility of 50% of population death exists due to inhalation of a toxic substance. LC1 region is a zone where a 1% possibility of death exists, and the IDLH region is a zone where reversible injuries following the inhalation of a toxic substance could occur. The safety limits for smoke pollutants are defined by the National Institute for Occupational Safety and Health (NIOSH) and for the zones LC1 and IDLH are 25,000 mg/m^3^ and 2500 mg/m^3^, respectively.

**Fig. 16:**
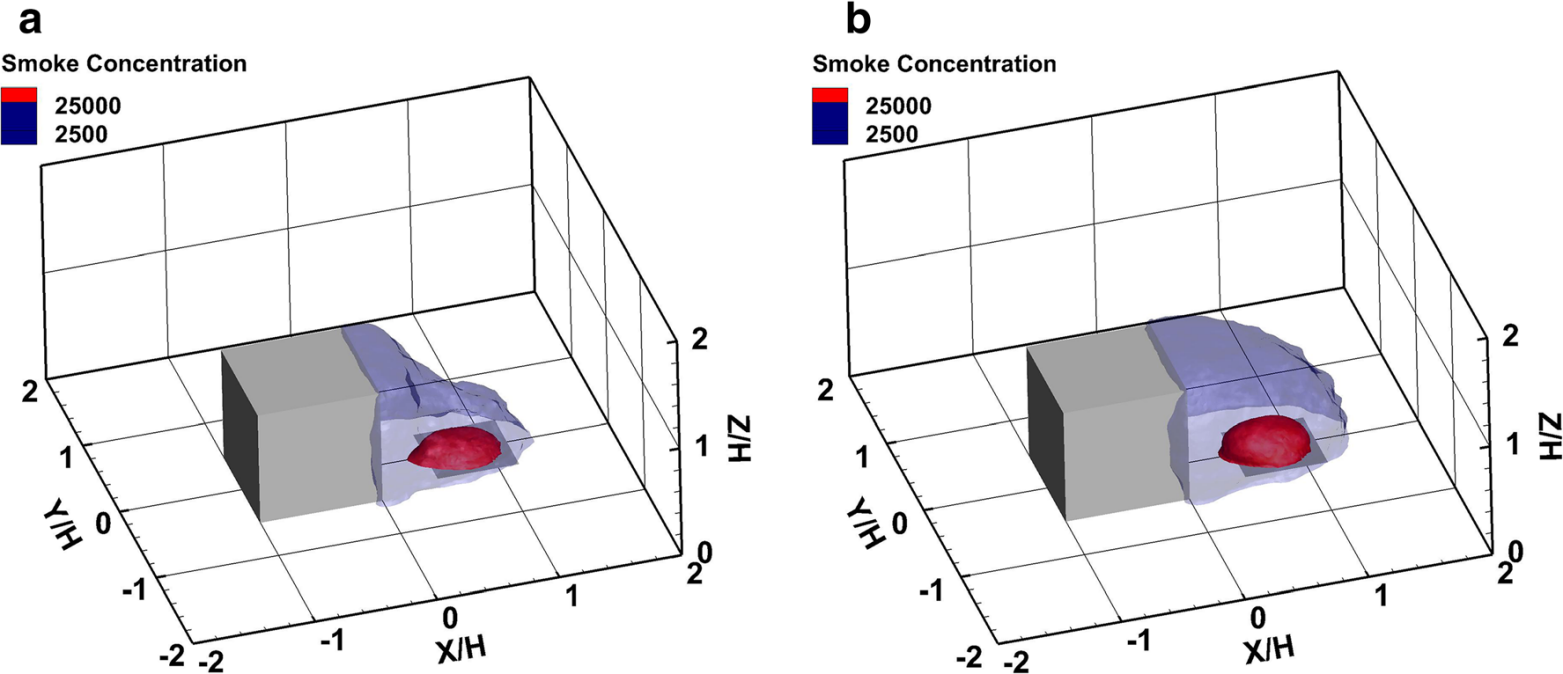
Iso-surfaces of mean smoke concentration (mg/m^3^) for the LC1 and IDLH zones, 200 s after the accident initiation. a Scenario 1. b Scenario 2

Figure 16 shows the safety limit zones for the two accident scenarios, 200 s after the initiation of the accident. The red color defines the boundaries of LC1 zone, and the blue color the boundaries of IDLH zone. The iso-surfaces of LC1 and IDLH zones assist in the visualization of the 3-D distribution of the hazardous released material after the initiation of the accident. In all scenarios, it is found that the hazardous material is transported by the buoyant plume and spreads towards the leeward face of the building. In both scenarios, the wake zone remains almost the same and the LC1 and IDLH toxic zones are limited within this zone.

**Fig. 17:**
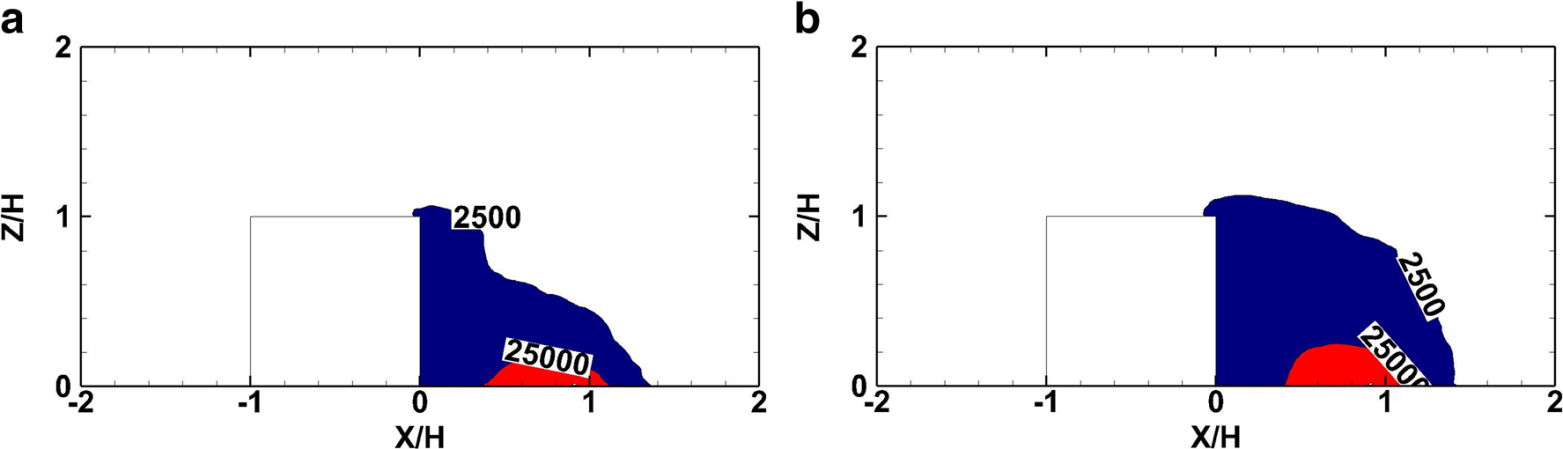
Iso-surfaces of smoke concentration (mg/m^3^) of the LC1 and IDLH zones. a Scenario 1. b Scenario 2

Figure 17 shows the LC1 and IDLH zones on the symmetry plane for both scenarios based on the mean smoke concentration. It is clear that from the beginning of the fire incident, the smoke is driven towards the leeward face of the cube, while the IDLH zone mainly affects the surface of the cube. A large portion of the smoke plume is trapped inside the recirculation zone making both accident scenarios to have almost the same toxic zone sizes. However, the dilution level inside the wake zone is not the same. In the diesel pool fire scenario, with larger smoke production, the IDLH zone covers the entire wake zone.

**Fig. 18:**
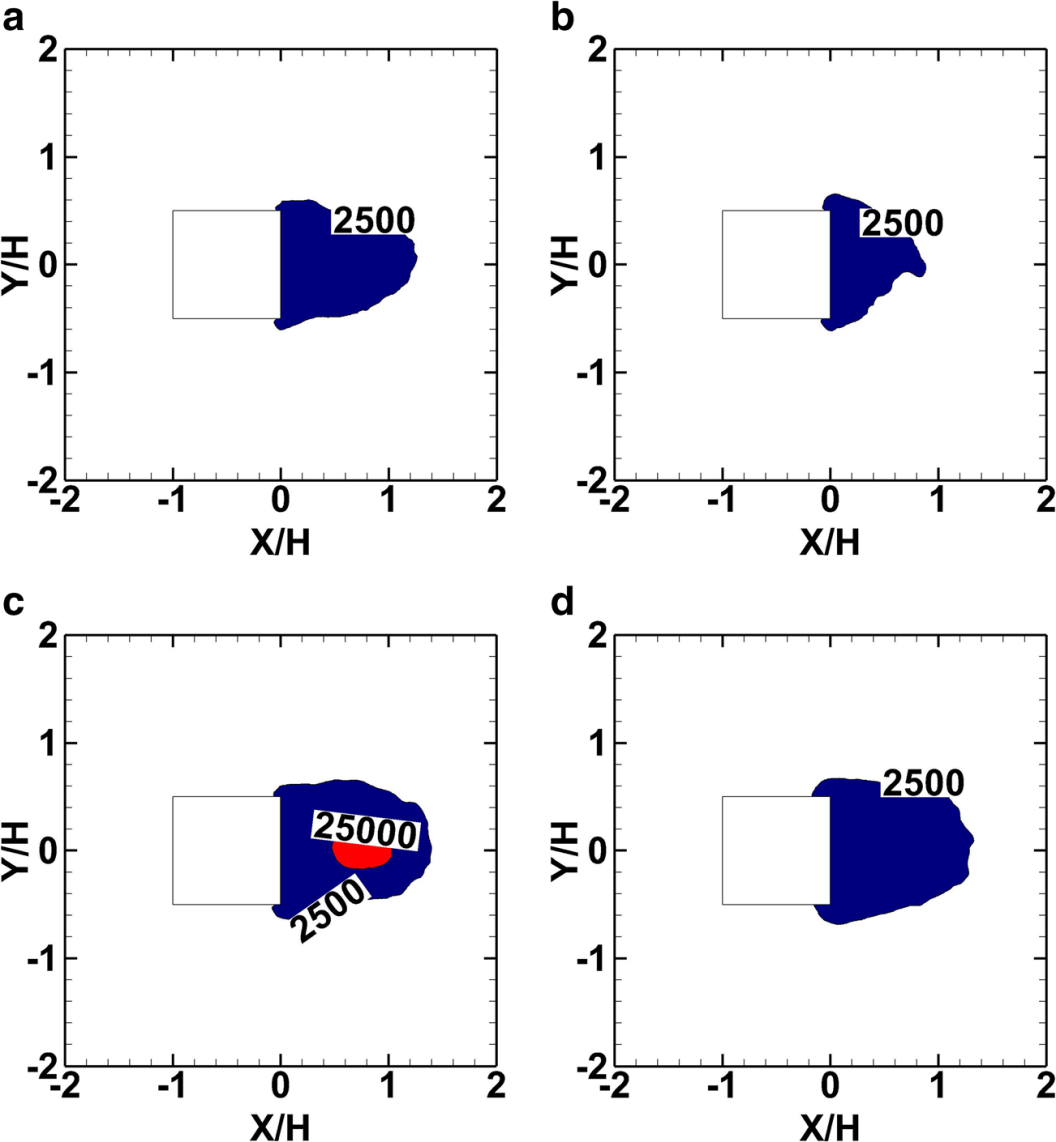
Iso-surface of the mean smoke concentration (mg/m^3^) of the LC1 and IDLH zones for scenario 1 and scenario 2. **a** Scenario 1, ***Z*** = 1m. **b** Scenario 1, ***Z*** = 3m. **c** Scenario 2, ***Z*** = 1m. **d** Scenario 2, ***Z*** = 3m

Figure 18 illustrates the IDLH and LC1 zones on the *Z*=1m and 3m horizontal planes for scenarios 1 and 2. Scenario 1 shows a significant IDLH zone area inside the cube cavity zone, which is getting smaller at higher heights (Fig. 18 a, b). Scenario 2 shows a significant smoke concentration at the height *Z*/H=1 (Fig. 18c) with both IDLH and LC1 zone areas limited within the cavity zone. At the higher level, *Z*/H=3, only the IDLH zone appears to have almost the same extent (Fig. 18d).

## Conclusions

The pollutant dispersion around a cubical building for two different pool fire scenarios, the crude oil fire and the diesel oil pool fire with 7.8 MW and 13.5 MW heat release rates, respectively, initiated in the cavity behind the building was studied by the LES method.

The numerical results for the wind flow around the cube fit successfully against the SILSOE cube field experimental data, where both the experiment and the present LES results are in agreement with respect to the wake reattachment length (*X*_*b*_ ≈ 1.4 *H*). The fire pollutant dispersion around the cube is also consistent with wind tunnel measurements. It was found that the strong buoyant forces (with Richardson numbers 2.36 and 2.56 for the crude oil and the diesel pool fires, respectively) and the turbulent mixing determine the extent of the toxic zones. The dispersion of smoke for the diesel pool fire shows important differences compared to that of the crude oil, i.e., higher smoke concentration inside the wake zone due to higher smoke generation, which can be about five times higher.

The smoke is trapped inside the boundaries of the cavity zone by the convective streamwise and wall-normal fluxes. The cavity zone, *X*_*b*_ , is 1.31 *H* for the crude oil pool fire and *X*_*b*_ 1.285 *H* for the diesel oil pool fire. The convective mass flux profiles on the symmetry plane are found similar for both accident scenarios, due to the common features of the turbulence field. The smoke generated by the fire is trapped by the dominant flow field of the recirculation region, which controls the extent of the toxic zones that are being approximately 1.4 Η in both fire scenarios. However, the dilution inside the wake area is not the same, because comparatively the diesel pool fire generates 65% more smoke which covers the entire cavity zone and with high concentration levels, so that the toxic zone extent at the symmetry plane becomes 60% larger. As a consequence, the toxic zone (IDLH) for the diesel pool fire covers almost the entire cavity zone, and only a small area is covered by the more toxic zone (based on LC1 index).

In the nutshell, it is concluded that the toxic zones of a fire accident may be defined accurately, and even though the two fire accidents studied release different amounts of heat, the impact on the toxic zones is similar, and the intervention methodologies can be the same.
